# Supplementary material for: SARS-CoV-2 serosurvey across multiple waves of the COVID-19 pandemic in New York City between 2020–2023
Source: Nat Commun. 2024 Jul 11;15:5847. doi: 10.1038/s41467-024-50052-2 (PMC11239669; doi:10.1038/s41467-024-50052-2)
Supplement: Supplementary file 1 — Supplementary Information [file 41467_2024_50052_MOESM1_ESM.pdf]

Supplementary information for:

SARS-CoV-2 serosurvey across multiple waves of the COVID-19 pandemic in New York City between 2020-2023

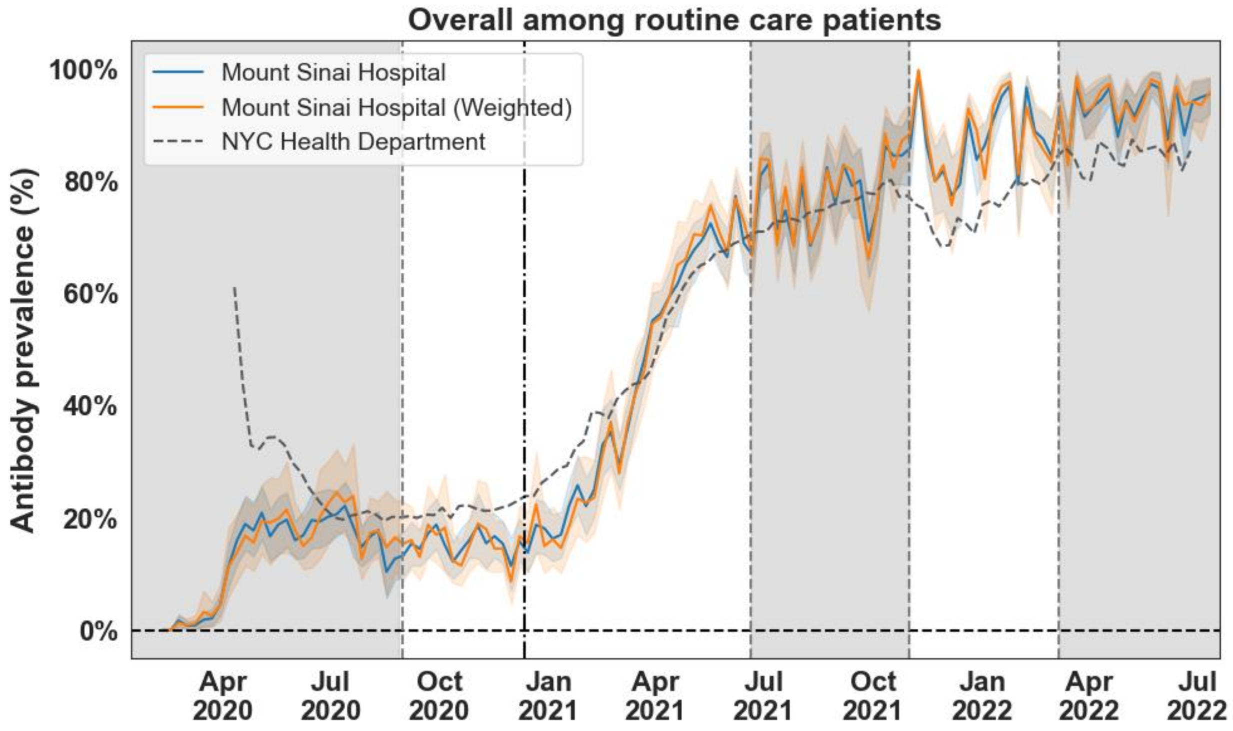

Supplementary Figure 1. SARS-CoV-2 mean spike binding antibody prevalence and titers in the Routine care and Urgent care groups at a Mount Sinai Hospital in New York City (NYC). Antibody prevalance in New York City reported by the NYC Department of Health and Mental Hygiene (NYC DOHMH) is represented by the dashed line. Antibody prevalence in the Routine Care group, measured between February 9th 2020 to July 18th 2022 with a 95% bootstrapped confidence interval. Weights are from Census 2022 estimates of the New York City population. Alternating shaded areas in all graphs denote the five successive epidemiological waves of infection in NYC. Grey color and vertical dashed lines serve as visual contrast.

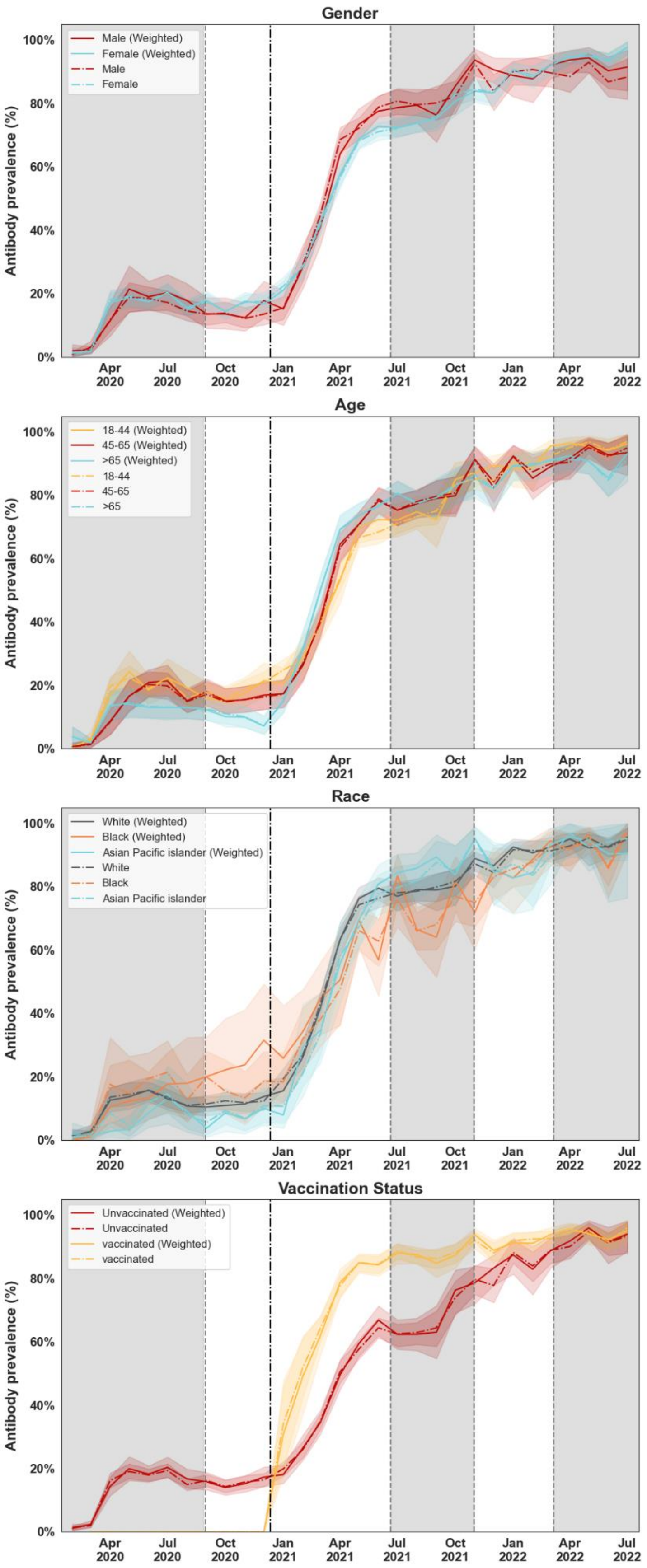

Supplementary Figure 2. SARS-CoV-2 spike antibody prevalence stratified by demographic groups and vaccination status. Antibody prevalence in the Routine Care groups, measured between February 9th 2020 to July 18th 2022, and stratified by gender, age, race, and vaccination status at the time of sample collection. Charts are fitted with a bootstrapped confidence interval based on random resampling of results to compute an estimate of the 95% confidence interval of the mean. Gender stratification: males, females. Categorical age levels: 18-44, 45-65, >65. Race stratification: White, Black, Asian and Pacific islander, Other, unknown. The date on which the first FDA-authorized SARS-CoV-2 vaccine became available in NYC is indicated by the vertical doted line and syringe. Vaccination status was assessed at time of sample collection and does not reflect vaccination rates in NYC or within our patient population. Weights are from Census 2022 estimates of the New York City population. Alternating shaded areas in all graphs denote the five successive epidemiological waves of infection in NYC. Grey color and vertical dashed lines serve as visual contrast.

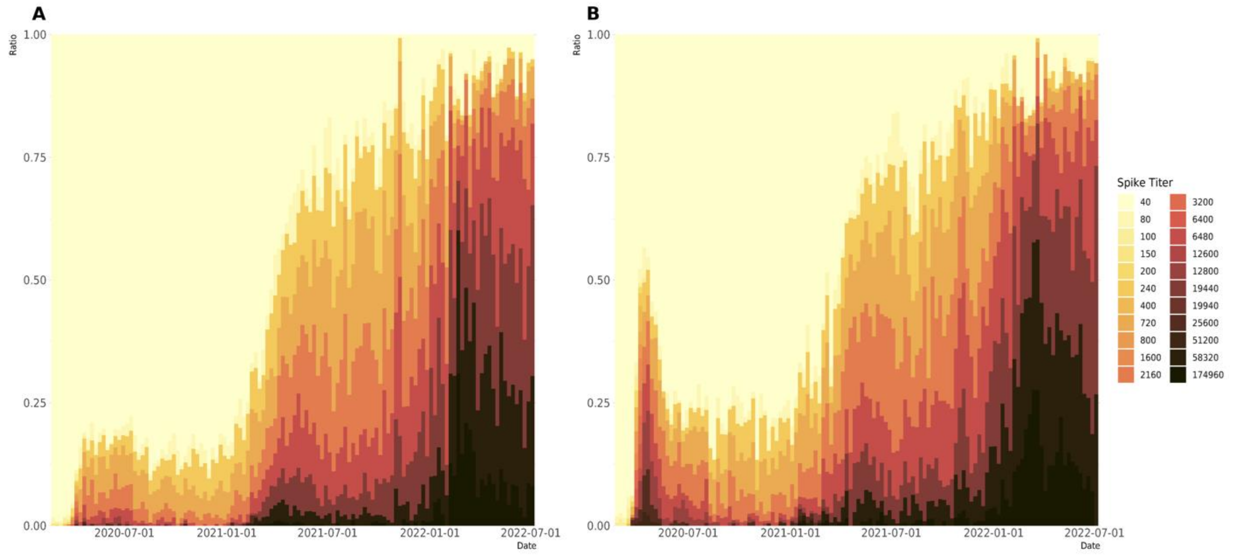

Supplementary Figure 3. Distribution of SARS-CoV-2 spike antibody titers during the serosurvey period. SARS-CoV-2 spike antibody titers in the Routine Care (left column), and Urgent care (right column) groups measured between February 9th 2020 to July 18th 2022. Weekly counts are presented per single column. Color gradient depicts the range of antibody titers.

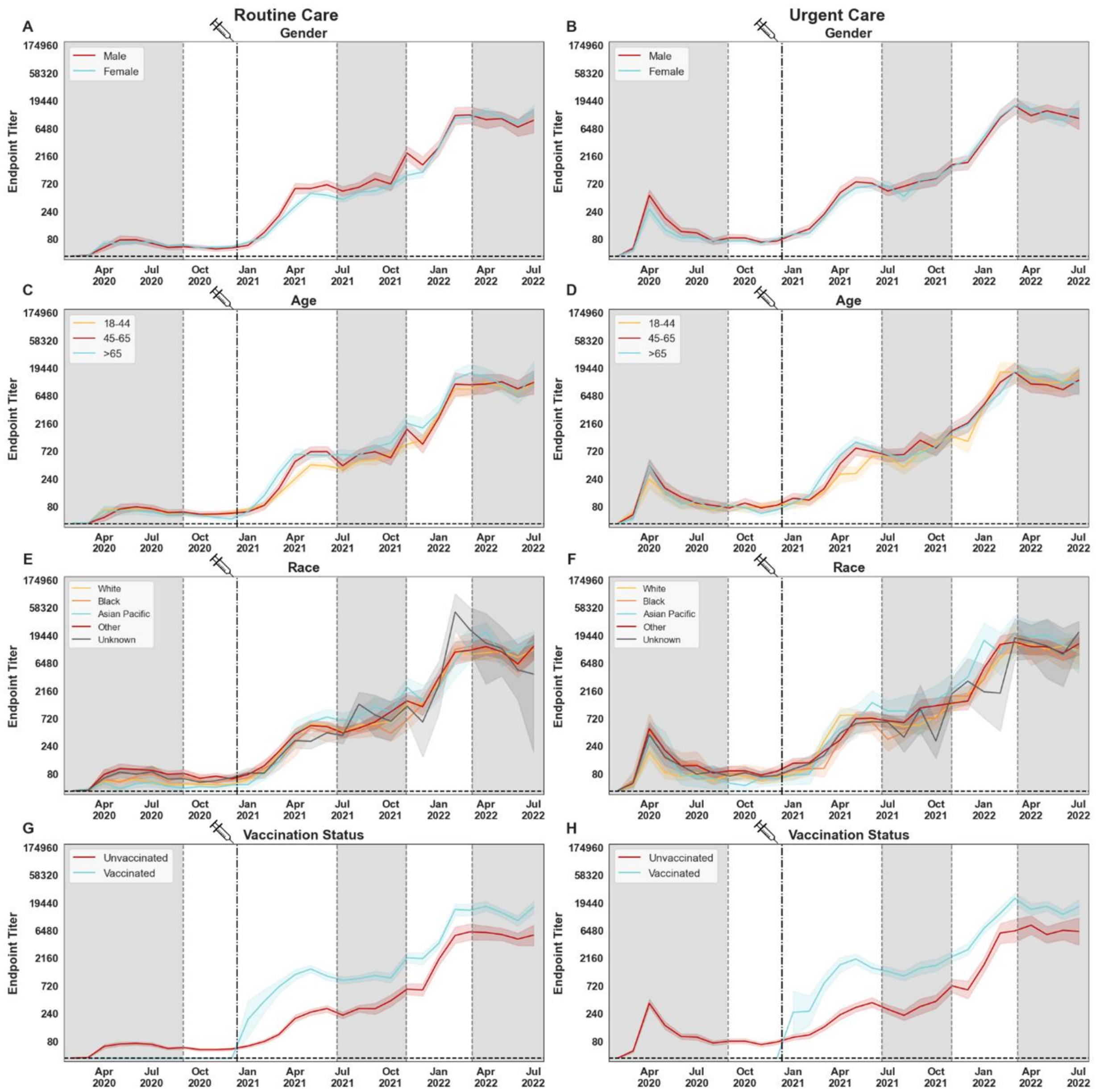

Supplementary Figure 4. SARS-CoV-2 spike antibody titers by demographic groups and vaccination status. SARS-CoV-2 spike antibody titers in the Routine Care (left column), and Urgent care (right column) groups measured between February 9th 2020 to July 18th 2022 stratified by gender (**A, B**), age (**C, D**), race (**E, F**), and vaccination status at the time of sample collection (**G, H**). Charts are fitted with a bootstrapped confidence interval based on random resampling of results to compute an estimate of the 95% confidence interval of the mean. **A, B.** Gender stratification: males, females. **C, D**. Categorical age levels: 18-44, 45-65, >65. Individuals <17 not shown in this analysis. **E, F**. Race stratification: White, Black, Asian and Pacific islander, Other, unknown. **G,H.** The date on which the first FDA-authorized SARS-CoV-2 vaccine became available in NYC is indicated by the vertical doted line and syringe. Vaccination status was assessed at time of sample collection and does not reflect vaccination rates in NYC or within our patient population. Alternating shaded areas in all graphs denote the five successive

epidemiological waves of infection in NYC. Grey color and vertical dashed lines serve as visual contrast.

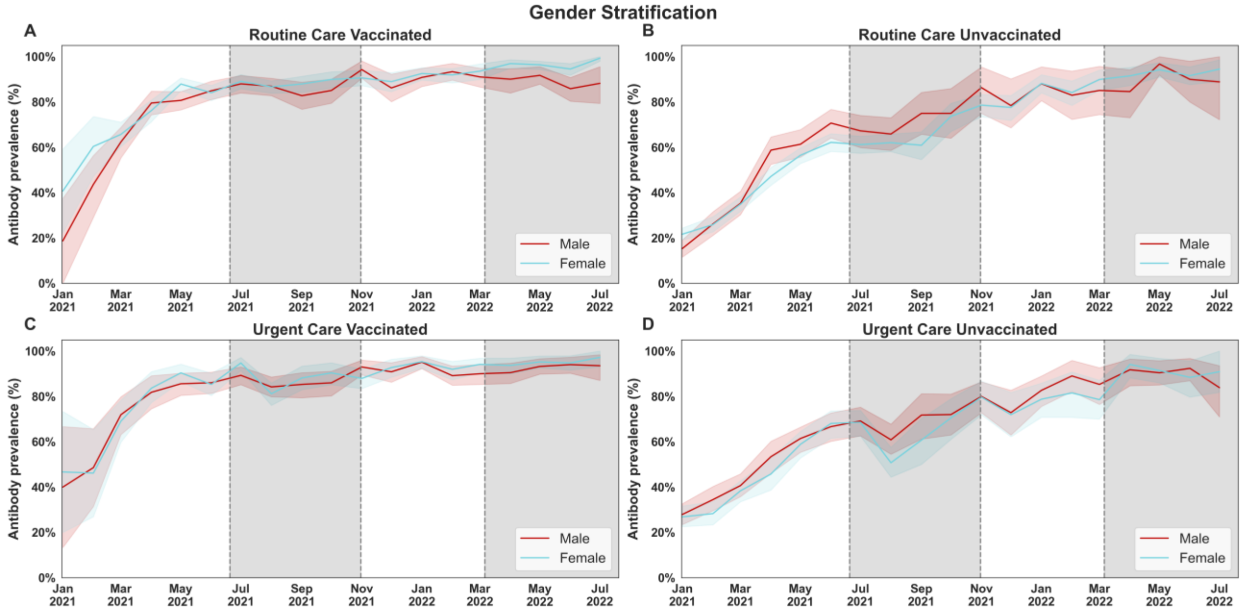

Supplementary Figure 5. SARS-CoV-2 spike antibody prevalence stratified by sex and vaccination status. Antibody prevalence in the Routine Care (top), and Urgent Care (bottom) groups, measured between February 9th 2020 to July 18th 2022, in vaccinated (left) and unvaccinated (right) inividuals. Charts are fitted with a bootstrapped confidence interval based on random resampling of results to compute an estimate of the 95% confidence interval of the mean. Vaccination status was assessed at time of sample collection and does not reflect vaccination rates in NYC or within our patient population. Alternating shaded areas in all graphs denote the five successive epidemiological waves of infection in NYC. Grey color and vertical dashed lines serve as visual contrast.

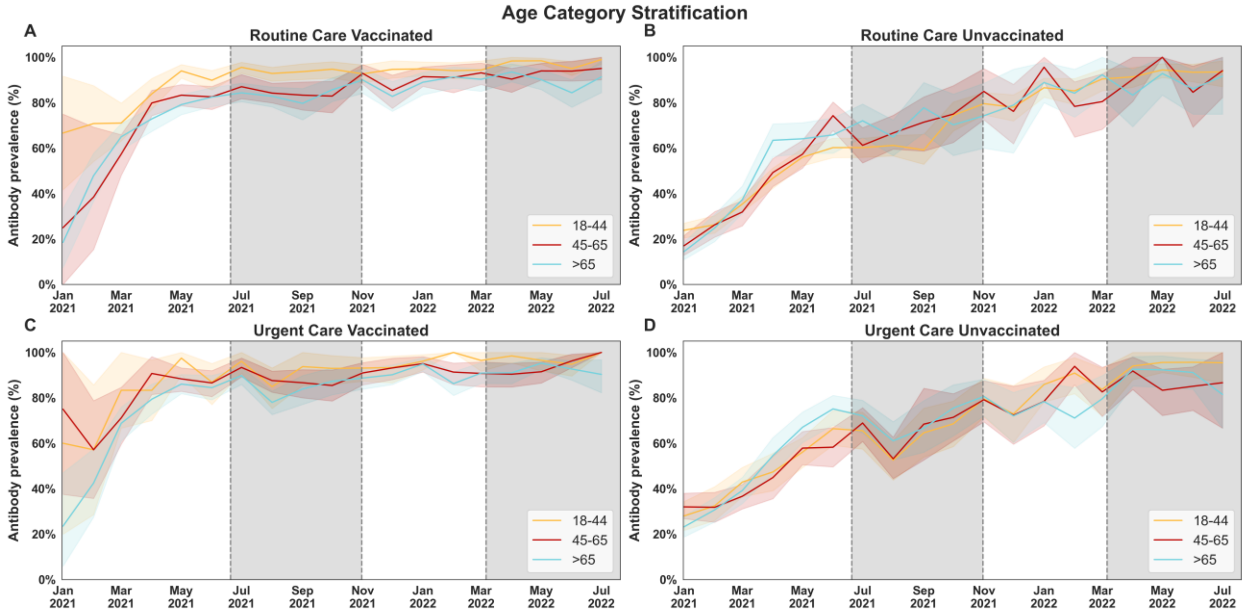

Supplementary Figure 6. SARS-CoV-2 spike antibody prevalence stratified by age and vaccination status. Antibody prevalence in the Routine Care (top), and Urgent Care (bottom) groups, measured between February 9th 2020 to July 18th 2022 in individuals with differen categorical age levels (18-44, 45-65, and >65 yeal old) in vaccinated (left) and unvaccinated (right) inividuals. Charts are fitted with a bootstrapped confidence interval based on random resampling of results to compute an estimate of the 95% confidence interval of the mean. Vaccination status was assessed at time of sample collection and does not reflect vaccination rates in NYC or within our patient population. Alternating shaded areas in all graphs denote the five successive epidemiological waves of infection in NYC. Grey color and vertical dashed lines serve as visual contrast.

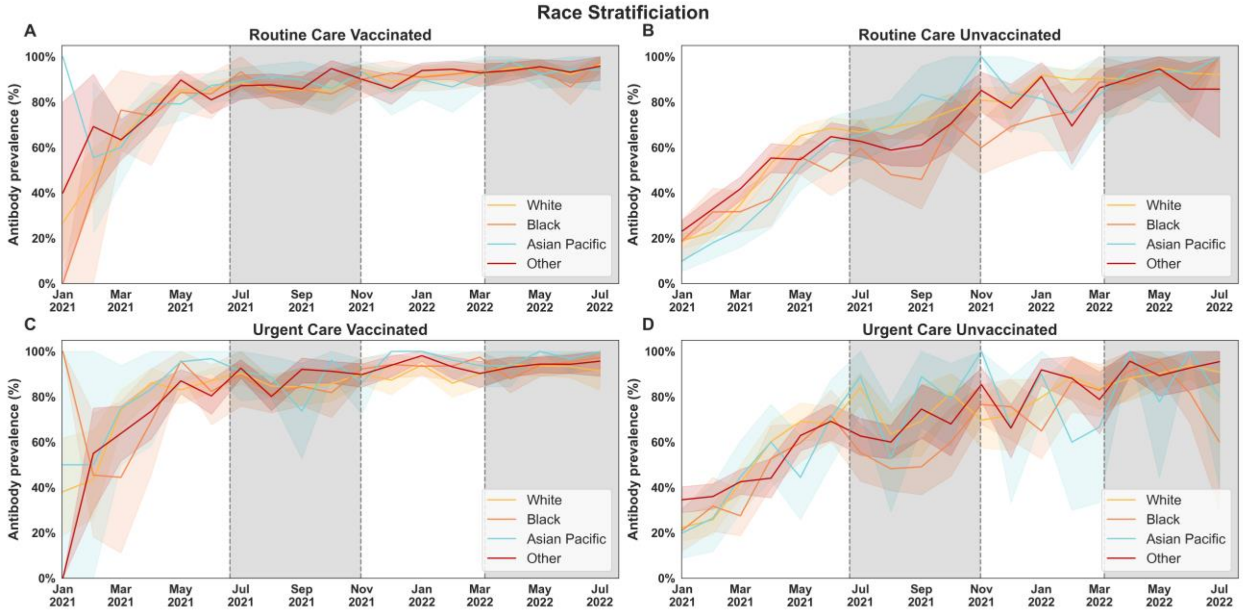

Supplementary Figure 7. SARS-CoV-2 spike antibody prevalence stratified by race and vaccination status. Antibody prevalence in the Routine Care (top), and Urgent Care (bottom) groups, measured between February 9th 2020 to July 18th 2022 in individuals of different racial categories (White, Black, Asian and Pacific islander, Other, unknown) in vaccinated (left) and unvaccinated (right) individuals. Charts are fitted with a bootstrapped confidence interval based on random resampling of results to compute an estimate of the 95% confidence interval of the mean. Vaccination status was assessed at time of sample collection and does not reflect vaccination rates in NYC or within our patient population. Alternating shaded areas in all graphs denote the five successive epidemiological waves of infection in NYC. Grey color and vertical dashed lines serve as visual contrast.

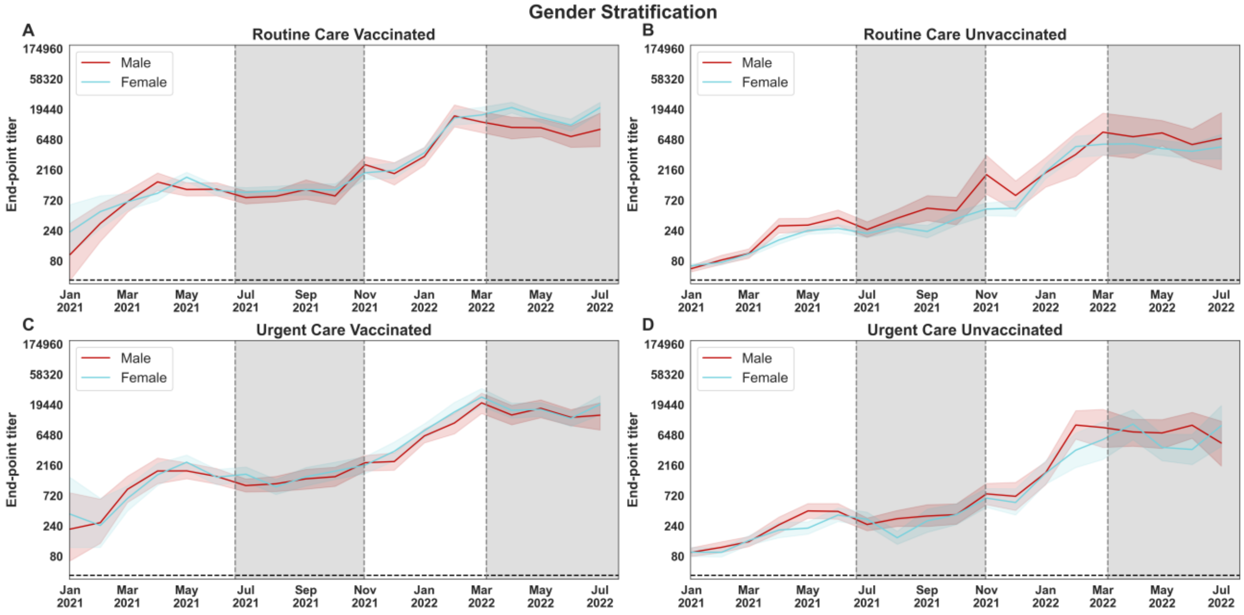

Supplementary Figure 8. SARS-CoV-2 anti-spike antibody titers stratified by sex and vaccination status. Antibody titers in the Routine Care (top), and Urgent Care (bottom) groups, measured between February 9th 2020 to July 18th 2022, in vaccinated (left) and unvaccinated (right) inividuals. Charts are fitted with a bootstrapped confidence interval based on random resampling of results to compute an estimate of the 95% confidence interval of the mean. Vaccination status was assessed at time of sample collection and does not reflect vaccination rates in NYC or within our patient population. Alternating shaded areas in all graphs denote the five successive epidemiological waves of infection in NYC. Grey color and vertical dashed lines serve as visual contrast.

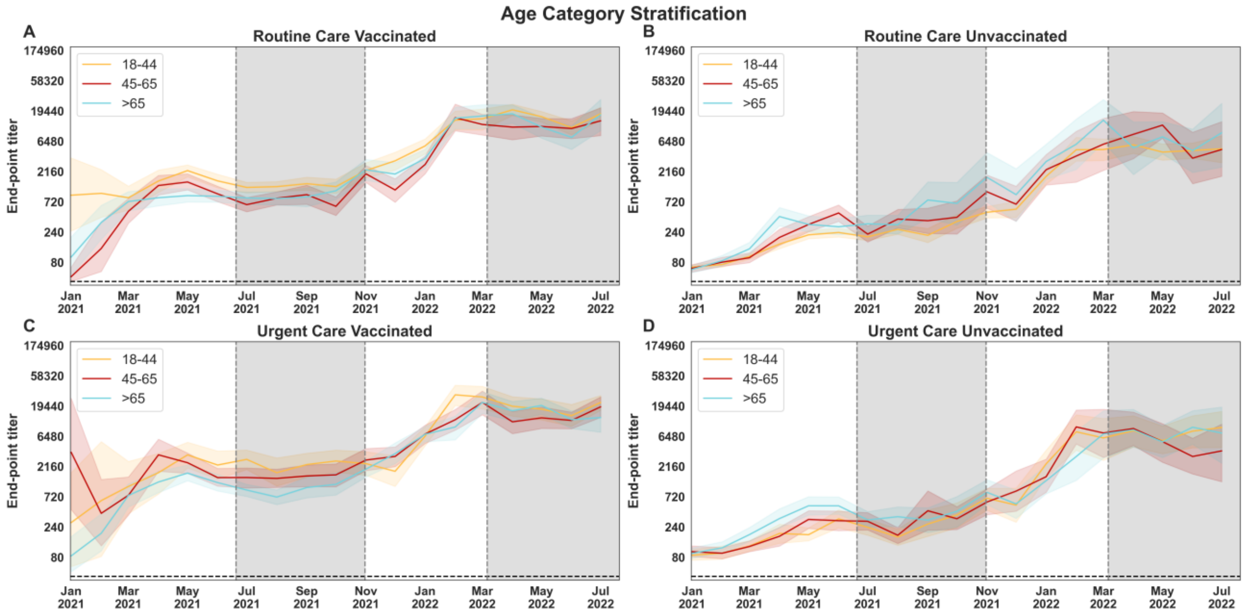

Supplementary Figure 9. SARS-CoV-2 anti-spike antibody titers stratified by age and vaccination status. Antibody titers in the Routine Care (top), and Urgent Care (bottom) groups, measured between February 9th 2020 to July 18th 2022 in individuals with differen categorical age levels (18-44, 45-65, and >65 yeal old) in vaccinated (left) and unvaccinated (right) inividuals. Charts are fitted with a bootstrapped confidence interval based on random resampling of results to compute an estimate of the 95% confidence interval of the mean. Vaccination status was assessed at time of sample collection and does not reflect vaccination rates in NYC or within our patient population. Alternating shaded areas in all graphs denote the five successive epidemiological waves of infection in NYC. Grey color and vertical dashed lines serve as visual contrast.

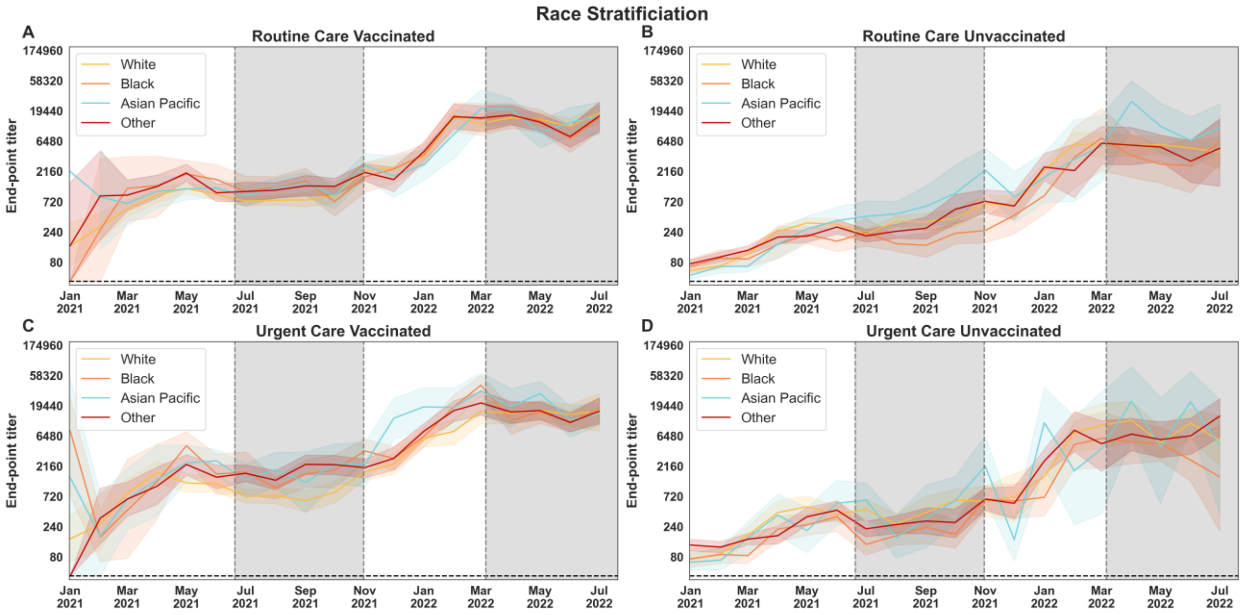

Supplementary Figure 10. SARS-CoV-2 anti-spike antibody titers stratified by race and vaccination status. Antibody titers in the Routine Care (top), and Urgent Care (bottom) groups, measured between February 9th 2020 to July 18th 2022 in individuals of different racial categories (White, Black, Asian and Pacific islander, Other, unknown) in vaccinated (left) and unvaccinated (right) individuals. Charts are fitted with a bootstrapped confidence interval based on random resampling of results to compute an estimate of the 95% confidence interval of the mean. Vaccination status was assessed at time of sample collection and does not reflect vaccination rates in NYC or within our patient population. Alternating shaded areas in all graphs denote the five successive epidemiological waves of infection in NYC. Grey color and vertical dashed lines serve as visual contrast.

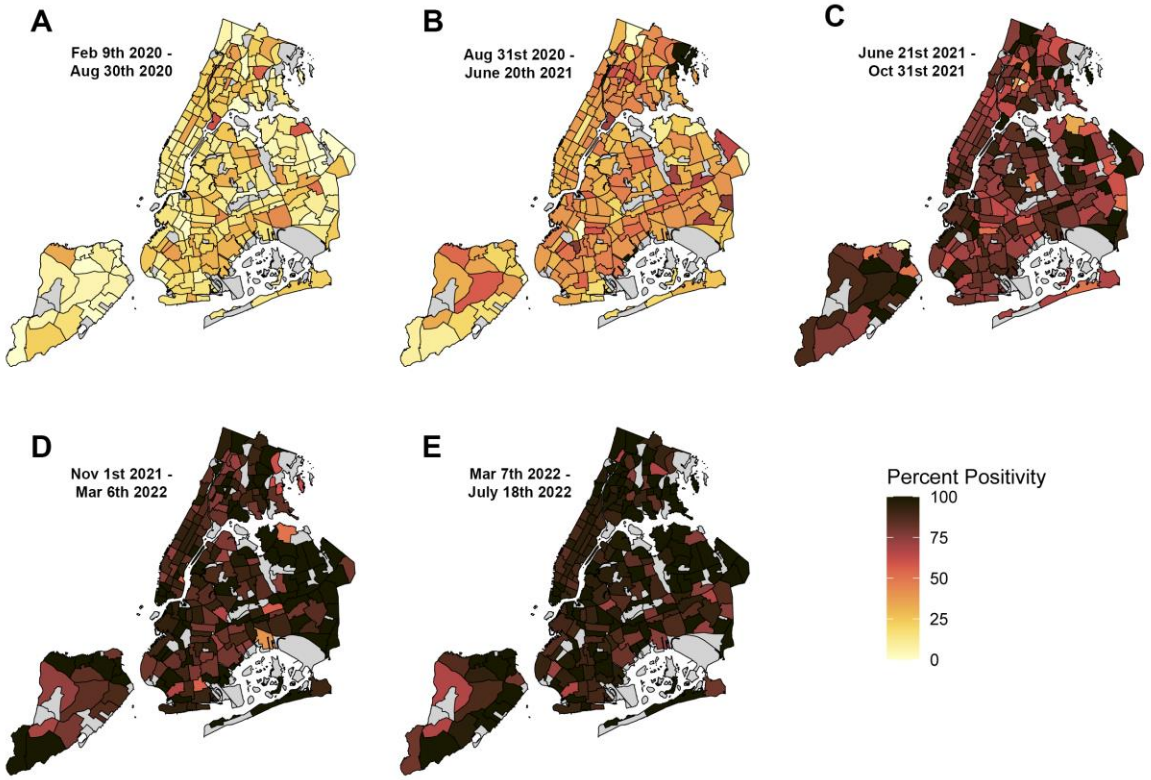

Supplementary Figure 11. Geographical distribution of SARS-CoV-2 spike antibody prevalence during five epidemiological waves of COVID-19 in residents of New York City (NYC). SARS-CoV-2 spike IgG prevalence measured in patients from the five boroughs of NYC (Manhattan, Brooklyn, Queens, The Bronx, and Staten Island, latitude and longitude: 40.7128° N, 74.0060° W) attending to a Mount Sinai Hospital in NYC are shown. 5 epidemiological waves corresponding to successive peaks of COVID-19 incidence in NYC are shown. **A**. Wave 1 (February 9th to August 30th, 2020). **B**. Wave 2 (August 31st, 2020, to June 20th, 2021). **C**. Wave 3 (June 21st to October 31st, 2021). **D**. Wave 4 (November 1st, 2021, to March 6th, 2022). **E**. Wave 5 (March 7th to July 18th, 2022). Antibody prevalence is expressed as percent positivity within neighborhood tabulation areas. Areas with less than 10 specimens are shaded grey. Color gradient depicts percent positivity (0 to 100%).

Supplementary Table 1. Distribution of demographic status and SARS-CoV-2 spike protein seropositivity among children <18 years old in a SARS-CoV-2 serosurveillance system in New York City.

	Urgent care group		Routine care group	
	Total (col. %)	S positive (row %)	Total (col. %)	S positive (row %)
Overall	354	166 (47%)	528	143 (27%)
Wave				
Wave 1 (through 30 Aug 2020)	114 (32%)	25 (22%)	293 (55%)	15 (5%)
Wave 2 (through 20 June 2021)	113 (32%)	33 (29%)	120 (23%)	33 (28%)
Wave 3 (through 31 Oct 2021)	48 (14%)	38 (79%)	48 (9%)	29 (60%)
Wave 4 (through 6 March 2022)	33 (9%)	28 (85%)	44 (8%)	43 (98%)
Wave 5 (through 18 July 2022)	46 (13%)	42 (91%)	23 (4%)	23 (100%)
Sex				
Female	181 (51%)	90 (50%)	272 (52%)	76 (28%)
Male	173 (49%)	76 (44%)	256 (48%)	67 (26%)
Race/ethnicity				
Asian Pacific Islander	20 (6%)	13 (65%)	46 (9%)	14 (30%)
Black	53 (15%)	36 (68%)	18 (3%)	14 (78%)
White	73 (21%)	26 (36%)	161 (30%)	54 (34%)
Other	145 (41%)	78 (54%)	179 (34%)	56 (31%)
Unknown	63 (18%)	13 (21%)	124 (23%)	5 (4%)
Insured				
Private	118 (33%)	49 (42%)	256 (48%)	69 (27%)
Public	213 (60%)	111 (52%)	219 (41%)	64 (29%)
Other	23 (7%)	6 (26%)	53 (10%)	10 (19%)
Vaccinated				
No	310 (88%)	123 (40%)	508 (96%)	124 (24%)
Yes	44 (12%)	43 (98%)	20 (4%)	19 (95%)

Supplementary Table 2. COVID-19 vaccination coverage over time among those receiving routine care in a SARS-CoV-2 serosurveillance system, New York City (N=33,489).

	Wave 1 (through 30 Aug 2020)	Wave 2 (through 20 June 2021)	Wave 3 (through 31 Oct 2021)	Wave 4 (through 6 March 2022)	Wave 5 (through 18 July 2022)
Overall	5/8634 (0%)	2314/13562 (17%)	2816/5359 (53%)	1714/2849 (60%)	2056/3085 (67%)
Age					
18-44 years	1/4293 (0%)	596/6506 (9%)	962/2597 (37%)	676/1509 (45%)	875/1594 (55%)
45-64 years	4/2134 (0%)	684/3418 (20%)	803/1276 (63%)	470/635 (74%)	547/692 (79%)
65+ years	0/2207 (0%)	1034/3638 (28%)	1051/1486 (71%)	568/705 (81%)	634/799 (79%)
Sex					
Female	5/5903 (0%)	1347/9326 (14%)	1747/3727 (47%)	1129/2055 (55%)	1355/2155 (63%)
Male	0/2730 (0%)	967/4236 (23%)	1069/1632 (66%)	585/794 (74%)	701/930 (75%)
Race/ethnicity					
Asian Pacific Islander	0/767 (0%)	296/1356 (22%)	330/524 (63%)	203/287 (71%)	241/315 (77%)
Black	0/386 (0%)	151/955 (16%)	331/662 (50%)	292/461 (63%)	374/494 (76%)
white	2/3721 (0%)	1122/5980 (19%)	1353/2447 (55%)	713/1314 (54%)	891/1509 (59%)
Other	0/2234 (0%)	529/3358 (16%)	662/1359 (49%)	488/744 (66%)	523/721 (73%)
Unknown	3/1526 (0%)	216/1913 (11%)	140/367 (38%)	18/43 (42%)	27/46 (59%)
Insured					
Private	4/4009 (0%)	933/6647 (14%)	1294/2626 (49%)	759/1466 (52%)	1000/1638 (61%)
Public	1/3960 (0%)	1239/6038 (21%)	1373/2418 (57%)	834/1176 (71%)	916/1232 (74%)
Other	0/665 (0%)	142/877 (16%)	149/315 (47%)	121/207 (58%)	140/215 (65%)

Supplementary Table 3. Demographic characteristics by epidemiological wave among those receiving routine care in a SARS-CoV-2 serosurveillance system, New York City (N=33,489).

		Wave 1 (through 30 Aug 2020)	Wave 2 (through 20 June 2021)	Wave 3 (through 31 Oct 2021)	Wave 4 (through 6 March 2022)	Wave 5 (through 18 July 2022)
Age	18-44 years	4293 (50%)	6506 (48%)	2597 (48%)	1509 (53%)	1594 (52%)
	45-64 years	2134 (25%)	3418 (25%)	1276 (24%)	635 (22%)	692 (22%)
	65+ years	2207 (26%)	3638 (27%)	1486 (28%)	705 (25%)	799 (26%)
Sex	Female	5903 (68%)	9326 (69%)	3727 (70%)	2055 (72%)	2155 (70%)
	Male	2730 (32%)	4236 (31%)	1632 (30%)	794 (28%)	930 (30%)
Race/ ethnicity	Asian Pacific Islander	767 (9%)	1356 (10%)	524 (10%)	287 (10%)	315 (10%)
	Black	386 (4%)	955 (7%)	662 (12%)	461 (16%)	494 (16%)
	white	3721 (43%)	5980 (44%)	2447 (46%)	1314 (46%)	1509 (49%)
	Other	2234 (26%)	3358 (25%)	1359 (25%)	744 (26%)	721 (23%)
	Unknown	1526 (18%)	1913 (14%)	367 (7%)	43 (2%)	46 (1%)
Insured	Private	4009 (46%)	6647 (49%)	2626 (49%)	1466 (51%)	1638 (53%)
	Public	3960 (46%)	6038 (45%)	2418 (45%)	1176 (41%)	1232 (40%)
	Other	665 (8%)	877 (6%)	315 (6%)	207 (7%)	215 (7%)
Vaccinated	No	8629 (100%)	11248 (83%)	2543 (47%)	1135 (40%)	1029 (33%)
	Yes	5 (0%)	2314 (17%)	2816 (53%)	1714 (60%)	2056 (67%)

Supplementary Table 4. Spike protein seropositivity among those receiving routine care in a SARS-CoV-2 serosurveillance system, New York City (N=33,489).

	Wave 1 (through 30 Aug 2020)	Wave 2 (through 20 June 2021)	Wave 3 (through 31 Oct 2021)	Wave 4 (through 6 March 2022)	Wave 5 (through 18 July 2022)
Overall	1116 (13%)	4909 (36%)	4081 (76%)	2502 (88%)	2871 (93%)
Age					
18-44 years	641 (15%)	2292 (35%)	1914 (74%)	1321 (88%)	1516 (95%)
45-64 years	260 (12%)	1265 (37%)	994 (78%)	565 (89%)	639 (92%)
65+ years	215 (10%)	1352 (37%)	1173 (79%)	616 (87%)	716 (90%)
Sex					
Female	793 (13%)	3320 (36%)	2773 (74%)	1791 (87%)	2038 (95%)
Male	323 (12%)	1589 (38%)	1308 (80%)	711 (90%)	833 (90%)
Race/ethnicity					
Asian Pacific Islander	56 (7%)	424 (31%)	428 (82%)	253 (88%)	295 (94%)
Black	53 (14%)	365 (38%)	457 (69%)	382 (83%)	456 (92%)
White	386 (10%)	2136 (36%)	1924 (79%)	1170 (89%)	1408 (93%)
Other	397 (18%)	1360 (41%)	1011 (74%)	658 (88%)	670 (93%)
Unknown	224 (15%)	624 (33%)	261 (71%)	39 (91%)	42 (91%)
Insured					
Private	503 (13%)	2400 (36%)	2044 (78%)	1295 (88%)	1536 (94%)
Public	536 (14%)	2183 (36%)	1798 (74%)	1026 (87%)	1133 (92%)
Other	77 (12%)	326 (37%)	239 (76%)	181 (87%)	202 (94%)
Vaccinated					
No	1116 (13%)	3131 (28%)	1638 (64%)	937 (83%)	942 (92%)
Yes	0 (0%)	1778 (77%)	2443 (87%)	1565 (91%)	1929 (94%)

Supplementary Table 5. Full parameter estimates from a multivariable Poisson regression of spike protein seropositivity among those receiving routine care in a SARS-CoV-2 serosurveillance system, New York City (N=33,488).

	PR (95% CI)	P-value for parameter estimate (two-tailed Z test)	P-value for likelihood ratio test (two-tailed chi-square test)
Wave			<0.0001
Wave 1 (through 30 Aug 2020)	ref		
Wave 2 (through 20 June 2021)	5.15 (4.26, 6.22)	<0.0001	
Wave 3 (through 31 Oct 2021)	11.51 (9.59, 13.81)	<0.0001	
Wave 4 (through 6 March 2022)	12.23 (10.19, 14.67)	<0.0001	
Wave 5 (through 18 July 2022)	12.64 (10.55, 15.13)	<0.0001	
Age			<0.0001
18-44 years	1.66 (1.41, 1.96)	<0.0001	
45-64 years	1.30 (1.09, 1.55)	0.0035	
65+ years	ref		
Sex			0.5882
Female	ref		
Male	1.04 (0.91, 1.19)	0.5865	
Race/ethnicity			<0.0001
Asian Pacific Islander	0.67 (0.51, 0.87)	0.0033	
Black	1.24 (0.94, 1.63)	0.1207	
white	ref		
Other	1.64 (1.44, 1.88)	<0.0001	
Unknown	1.33 (1.14, 1.56)	0.0004	
Insured			0.0258
Private	ref		
Public	1.14 (1.00, 1.29)	0.0432	
Other	0.88 (0.70, 1.11)	0.2882	
Age*Wave			<0.0001

18-44 years*Wave 2	0.57 (0.47, 0.68)	<0.0001	
18-44 years*Wave 3	0.56 (0.47, 0.66)	<0.0001	
18-44 years*Wave 4	0.61 (0.51, 0.72)	<0.0001	
18-44 years*Wave 5	0.63 (0.53, 0.75)	<0.0001	
45-64 years*Wave 2	0.75 (0.62, 0.91)	0.0028	
45-64 years*Wave 3	0.75 (0.63, 0.90)	0.0019	
45-64 years*Wave 4	0.79 (0.66, 0.94)	0.0089	
45-64 years*Wave 5	0.80 (0.67, 0.95)	0.0131	
Sex*Wave			0.0001
Male*Wave 2	1.00 (0.86, 1.15)	0.9678	
Male*Wave 3	1.02 (0.89, 1.17)	0.7941	
Male*Wave 4	0.99 (0.86, 1.14)	0.8958	
Male*Wave 5	0.92 (0.81, 1.06)	0.2630	
Race/ethnicity *Wave			<0.0001
Asian Pacific Islander*Wave 2	1.31 (0.99, 1.74)	0.0596	
Asian Pacific Islander*Wave 3	1.56 (1.19, 2.05)	0.0013	
Asian Pacific Islander*Wave 4	1.48 (1.13, 1.94)	0.0048	
Asian Pacific Islander*Wave 5	1.51 (1.15, 1.98)	0.0029	
Black*Wave 2	0.87 (0.65, 1.15)	0.3292	
Black*Wave 3	0.72 (0.54, 0.95)	0.0200	
Black*Wave 4	0.75 (0.57, 0.99)	0.0416	
Black*Wave 5	0.79 (0.60, 1.05)	0.1004	
Other*Wave 2	0.70 (0.60, 0.80)	<0.0001	
Other*Wave 3	0.59 (0.51, 0.67)	<0.0001	
Other*Wave 4	0.60 (0.52, 0.69)	<0.0001	
Other*Wave 5	0.61 (0.53, 0.70)	<0.0001	
Unknown*Wave 2	0.69 (0.58, 0.83)	<0.0001	
Unknown*Wave 3	0.69 (0.58, 0.82)	<0.0001	
Unknown*Wave 4	0.77 (0.64, 0.92)	0.0053	
Unknown*Wave 5	0.73 (0.61, 0.87)	0.0005	

Insured*Wave			0.0018
Public*Wave 2	0.85 (0.74, 0.97)	0.0163	
Public*Wave 3	0.82 (0.72, 0.93)	0.0021	
Public*Wave 4	0.87 (0.77, 0.99)	0.0402	
Public*Wave 5	0.89 (0.78, 1.01)	0.0716	
Other*Wave 2	1.15 (0.90, 1.47)	0.2736	
Other*Wave 3	1.11 (0.87, 1.40)	0.4013	
Other*Wave 4	1.12 (0.89, 1.42)	0.3338	
Other*Wave 5	1.14 (0.90, 1.44)	0.2719	

Supplementary Table 6. Multivariable Poisson regression of spike protein seropositivity among the unvaccinated in routine care visits in a SARS-CoV-2 serosurveillance system, New York City (N=24,584).

	Wave 1 (through 30 Aug 2020)	Wave 2 (through 20 June 2021)	Wave 3 (through 31 Oct 2021)	Wave 4 (through 6 March 2022)	Wave 5 (through 18 July 2022)
	PR (95% CI)	PR (95% CI)	PR (95% CI)	PR (95% CI)	PR (95% CI)
Age					
18-44 years	**1.66 (1.41, 1.96)**	**1.26 (1.15, 1.39)**	**0.90 (0.83, 0.99)**	1.03 (0.93, 1.14)	1.05 (0.97, 1.12)
45-64 years	**1.30 (1.09, 1.55)**	**1.12 (1.02, 1.24)**	0.98 (0.89, 1.08)	1.07 (0.95, 1.19)	1.01 (0.92, 1.10)
65+ years	ref	ref	ref	ref	ref
Sex					
Female	ref	ref	ref	ref	ref
Male	1.04 (0.91, 1.19)	1.04 (0.97, 1.12)	1.05 (0.98, 1.14)	1.02 (0.95, 1.10)	0.99 (0.94, 1.05)
Race/ethnicity					
Asian Pacific Islander	**0.67 (0.51, 0.87)**	**0.70 (0.61, 0.80)**	1.01 (0.91, 1.11)	1.00 (0.91, 1.09)	1.00 (0.93, 1.08)
Black	1.24 (0.94, 1.63)	**1.15 (1.03, 1.29)**	**0.77 (0.69, 0.87)**	**0.78 (0.70, 0.88)**	0.98 (0.92, 1.05)
white	ref	ref	ref	ref	ref
Other	**1.64 (1.44, 1.88)**	**1.26 (1.17, 1.35)**	0.93 (0.87, 1.00)	0.95 (0.89, 1.02)	0.97 (0.92, 1.02)
Unknown	**1.33 (1.14, 1.56)**	1.02 (0.93, 1.11)	**0.87 (0.78, 0.98)**	1.02 (0.88, 1.19)	1.02 (0.92, 1.14)
Insured					
Private	ref	ref	ref	ref	ref
Public	**1.14 (1.00, 1.29)**	0.95 (0.89, 1.02)	**0.92 (0.86, 0.99)**	1.02 (0.95, 1.10)	1.01 (0.96, 1.07)
Other	0.88 (0.70, 1.11)	1.01 (0.90, 1.14)	0.96 (0.84, 1.09)	0.96 (0.85, 1.07)	0.99 (0.92, 1.07)

Note: Full list of p-values in Supplementary Table 7.

Supplementary Table 7. Full parameter estimates from a multivariable Poisson regression of spike protein seropositivity among the unvaccinated in routine care visits in a SARS-CoV-2 serosurveillance system, New York City (N=24,584).

	PR (95% CI)	P-value for parameter estimate (two-tailed Z test)	P-value for likelihood ratio test (two-tailed chi-square test)
Wave			<0.0001
Wave 1 (through 30 Aug 2020)	ref		
Wave 2 (through 20 June 2021)	3.18 (2.60, 3.89)	<.0001	
Wave 3 (through 31 Oct 2021)	10.40 (8.52, 12.70)	<.0001	
Wave 4 (through 6 March 2022)	11.55 (9.41, 14.16)	<.0001	
Wave 5 (through 18 July 2022)	12.37 (10.21, 14.99)	<.0001	
Age			<0.0001
18-44 years	1.66 (1.41, 1.96)	<.0001	
45-64 years	1.30 (1.09, 1.55)	0.0033	
65+ years	ref		
Sex			0.6008
Female	ref		
Male	1.04 (0.91, 1.19)	0.5992	
Race/ethnicity			<0.0001
Asian Pacific Islander	0.67 (0.51, 0.87)	0.0033	
Black	1.24 (0.94, 1.63)	0.1216	
white	ref		
Other	1.64 (1.44, 1.88)	<.0001	
Unknown	1.33 (1.14, 1.56)	0.0004	
Insured			0.0263
Private	ref		
Public	1.14 (1.00, 1.29)	0.0445	
Other	0.88 (0.70, 1.11)	0.2852	
Age*Wave			<0.0001

18-44 years*Wave 2	0.76 (0.63, 0.92)	0.0047	
18-44 years*Wave 3	0.54 (0.45, 0.66)	<.0001	
18-44 years*Wave 4	0.62 (0.51, 0.75)	<.0001	
18-44 years*Wave 5	0.63 (0.53, 0.75)	<.0001	
45-64 years*Wave 2	0.86 (0.71, 1.05)	0.1503	
45-64 years*Wave 3	0.75 (0.62, 0.92)	0.0059	
45-64 years*Wave 4	0.82 (0.67, 1.01)	0.0617	
45-64 years*Wave 5	0.77 (0.64, 0.94)	0.0102	
Sex*Wave			0.7752
Male*Wave 2	1.00 (0.86, 1.17)	0.9536	
Male*Wave 3	1.02 (0.87, 1.19)	0.8377	
Male*Wave 4	0.98 (0.84, 1.15)	0.8419	
Male*Wave 5	0.96 (0.83, 1.11)	0.5768	
Race/ethnicity *Wave			<0.0001
Asian Pacific Islander*Wave 2	1.04 (0.77, 1.41)	0.7849	
Asian Pacific Islander*Wave 3	1.51 (1.13, 2.01)	0.0050	
Asian Pacific Islander*Wave 4	1.49 (1.12, 1.98)	0.0061	
Asian Pacific Islander*Wave 5	1.50 (1.14, 1.98)	0.0042	
Black*Wave 2	0.93 (0.69, 1.25)	0.6310	
Black*Wave 3	0.62 (0.46, 0.84)	0.0018	
Black*Wave 4	0.63 (0.47, 0.85)	0.0022	
Black*Wave 5	0.79 (0.60, 1.05)	0.1050	
Other*Wave 2	0.76 (0.66, 0.89)	0.0006	
Other*Wave 3	0.57 (0.49, 0.66)	<.0001	
Other*Wave 4	0.58 (0.50, 0.68)	<.0001	
Other*Wave 5	0.59 (0.51, 0.68)	<.0001	
Unknown*Wave 2	0.76 (0.64, 0.92)	0.0039	
Unknown*Wave 3	0.66 (0.54, 0.80)	<.0001	
Unknown*Wave 4	0.77 (0.62, 0.96)	0.0188	
Unknown*Wave 5	0.77 (0.63, 0.93)	0.0067	

Insured*Wave			0.0612
Public*Wave 2	0.84 (0.73, 0.97)	0.0146	
Public*Wave 3	0.81 (0.70, 0.94)	0.0044	
Public*Wave 4	0.90 (0.78, 1.04)	0.1546	
Public*Wave 5	0.89 (0.78, 1.02)	0.1003	
Other*Wave 2	1.15 (0.89, 1.49)	0.2924	
Other*Wave 3	1.09 (0.84, 1.41)	0.5386	
Other*Wave 4	1.08 (0.84, 1.40)	0.5400	
Other*Wave 5	1.13 (0.88, 1.43)	0.3385	

Supplementary Table 8. Multivariable Poisson regression of spike protein seropositivity among those receiving routine care in a SARS-CoV-2 serosurveillance system, New York City (N=19,168).

	Wave 2 (January 11[a] through 20 June 2021)	Wave 3 (through 31 Oct 2021)	Wave 4 (through 6 March 2022)	Wave 5 (through 18 July 2022)	*p*-value[b]
	PR (95% CI)	PR (95% CI)	PR (95% CI)	PR (95% CI)	
Age					0.7440
18-44 years	**1.07 (1.01, 1.13)**	1.02 (0.98, 1.07)	**1.05 (1.01, 1.09)**	**1.06 (1.03, 1.09)**	
45-64 years	1.01 (0.95, 1.07)	1.00 (0.96, 1.04)	1.03 (0.98, 1.07)	**1.04 (1.00, 1.07)**	
65+ years	ref	ref	ref	ref	
Sex					0.0001
Female	ref	ref	ref	ref	
Male	1.03 (0.99, 1.08)	**1.05 (1.01, 1.08)**	1.03 (0.99, 1.06)	**0.96 (0.94, 0.99)**	
Race/ethnicity					<.0001
Asian Pacific Islander	**0.86 (0.80, 0.93)**	1.02 (0.98, 1.07)	0.97 (0.93, 1.02)	1.00 (0.97, 1.04)	
Black	1.00 (0.92, 1.09)	**0.90 (0.85, 0.95)**	**0.92 (0.88, 0.97)**	0.98 (0.95, 1.01)	
White	ref	ref	ref	ref	
Other	**1.05 (1.00, 1.11)**	0.97 (0.94, 1.01)	0.98 (0.95, 1.01)	0.99 (0.97, 1.02)	
Unknown	0.94 (0.88, 1.01)	0.96 (0.90, 1.02)	1.03 (0.93, 1.13)	0.97 (0.89, 1.05)	
Insured					0.0509
Private	ref	ref	ref	ref	
Public	0.96 (0.91, 1.00)	**0.94 (0.91, 0.97)**	0.99 (0.96, 1.02)	1.01 (0.99, 1.04)	
Other	1.00 (0.92, 1.09)	0.98 (0.92, 1.05)	0.99 (0.94, 1.04)	1.01 (0.97, 1.04)	
Vaccinated					<.0001
No	ref	ref	ref	ref	
Yes	**1.95 (1.87, 2.03)**	**1.35 (1.30, 1.39)**	**1.12 (1.09, 1.16)**	**1.04 (1.02, 1.07)**	

Notes: PR: prevalence ratio; CI: confidence interval

[a] Earlier dates excluded. Analysis limited to the time frame where vaccine was available (N=19,168).

[a] a p-value for interaction term between wave and characteristic according to a two-tailed chi-square type 3 analysis. Significant values indicate substantial changes in PRs across waves for a specific characteristic. Full list of p-values in Supplementary Table 9.

Supplementary Table 9. Full parameter estimates from a multivariable Poisson regression of spike protein seropositivity among those receiving routine care in a SARS-CoV-2 serosurveillance system, New York City (N=19,168).

	PR (95% CI)	P-value for parameter estimate (two-tailed Z test)	P-value for likelihood ratio test (two-tailed chi-square test)
Wave			<0.0001
Wave 2 (through 20 June 2021)	Ref		
Wave 3 (through 31 Oct 2021)	1.67 (1.53, 1.82)	<.0001	
Wave 4 (through 6 March 2022)	2.05 (1.88, 2.23)	<.0001	
Wave 5 (through 18 July 2022)	2.24 (2.07, 2.42)	<.0001	
Age			0.0548
18-44 years	1.07 (1.01, 1.13)	0.0311	
45-64 years	1.01 (0.95, 1.07)	0.7530	
65+ years	ref		
Sex			0.1669
Female	ref		
Male	1.03 (0.99, 1.08)	0.1658	
Race/ethnicity			<0.0001
Asian Pacific Islander	0.86 (0.80, 0.93)	<.0001	
Black	1.00 (0.92, 1.09)	0.9537	
white	ref		
Other	1.05 (1.00, 1.11)	0.0391	
Unknown	0.94 (0.88, 1.01)	0.0853	
Insured			0.1726
Private	ref		
Public	0.96 (0.91, 1.00)	0.0752	
Other	1.00 (0.92, 1.09)	0.9135	
Vaccinated			<0.0001
No	ref		
Yes	1.95 (1.87, 2.03)	<.0001	

Age*Wave			0.7440
18-44 years*Wave 3	0.96 (0.89, 1.03)	0.2628	
18-44 years*Wave 4	0.98 (0.91, 1.05)	0.5951	
18-44 years*Wave 5	0.99 (0.93, 1.06)	0.8488	
45-64 years*Wave 3	0.99 (0.92, 1.06)	0.7806	
45-64 years*Wave 4	1.02 (0.95, 1.09)	0.6273	
45-64 years*Wave 5	1.03 (0.96, 1.10)	0.4366	
Sex*Wave			0.0001
Male*Wave 3	1.01 (0.95, 1.07)	0.6861	
Male*Wave 4	0.99 (0.94, 1.05)	0.8251	
Male*Wave 5	0.93 (0.88, 0.98)	0.0068	
Race/ethnicity *Wave			<0.0001
Asian Pacific Islander*Wave 3	1.18 (1.08, 1.29)	0.0001	
Asian Pacific Islander*Wave 4	1.13 (1.03, 1.23)	0.0066	
Asian Pacific Islander*Wave 5	1.16 (1.07, 1.26)	0.0003	
Black*Wave 3	0.90 (0.82, 0.99)	0.0303	
Black*Wave 4	0.92 (0.84, 1.01)	0.0919	
Black*Wave 5	0.98 (0.90, 1.07)	0.6093	
Other*Wave 3	0.92 (0.87, 0.98)	0.0115	
Other*Wave 4	0.93 (0.87, 0.99)	0.0164	
Other*Wave 5	0.94 (0.89, 1.00)	0.0381	
Unknown*Wave 3	1.02 (0.93, 1.12)	0.6695	
Unknown*Wave 4	1.09 (0.97, 1.23)	0.1574	
Unknown*Wave 5	1.03 (0.92, 1.15)	0.6116	
Insured*Wave			0.0509
Public*Wave 3	0.98 (0.93, 1.04)	0.5805	
Public*Wave 4	1.03 (0.97, 1.10)	0.2882	
Public*Wave 5	1.06 (1.00, 1.12)	0.0473	
Other*Wave 3	0.98 (0.88, 1.09)	0.6907	
Other*Wave 4	0.98 (0.89, 1.09)	0.7394	

Other*Wave 5	1.00 (0.91, 1.10)	0.9937	
Vaccinated*Wave			<0.0001
Vaccinated*Wave 3	0.69 (0.66, 0.73)	<.0001	
Vaccinated*Wave 4	0.58 (0.55, 0.61)	<.0001	
Vaccinated*Wave 5	0.53 (0.51, 0.56)	<.0001	

Supplementary Table 10. Multivariable Poisson regression of spike protein seropositivity in a SARS-CoV-2 serosurveillance system, adults 18-64, New York City (N=24,653).

	Wave 1 (through 30 Aug 2020)	Wave 2 (through 20 June 2021)	Wave 3 (through 31 Oct 2021)	Wave 4 (through 6 March 2022)	Wave 5 (through 18 July 2022)	*p*-value[a]
	PR (95% CI)	PR (95% CI)	PR (95% CI)	PR (95% CI)	PR (95% CI)	
Age						0.0013
18-44 years	**1.26 (1.09, 1.46)**	0.96 (0.90, 1.02)	0.96 (0.92, 1.01)	0.99 (0.95, 1.02)	1.02 (0.99, 1.04)	
45-64 years	ref	ref	ref	ref	ref	
Sex						0.0035
Female	ref	ref	ref	ref	ref	
Male	1.01 (0.86, 1.19)	1.02 (0.96, 1.10)	**1.09 (1.04, 1.14)**	1.03 (0.99, 1.07)	0.97 (0.95, 1.00)	
Race/ethnicity						<.0001
Asian Pacific Islander	**0.67 (0.51, 0.89)**	**0.80 (0.72, 0.89)**	**1.07 (1.02, 1.13)**	1.02 (0.98, 1.07)	1.01 (0.98, 1.05)	
Black	1.27 (0.95, 1.69)	1.05 (0.95, 1.16)	**0.89 (0.84, 0.95)**	**0.91 (0.86, 0.97)**	1.01 (0.98, 1.04)	
White	ref	ref	ref	ref	ref	
Other	**1.55 (1.33, 1.81)**	**1.12 (1.05, 1.20)**	**0.95 (0.91, 1.00)**	0.97 (0.93, 1.01)	0.99 (0.97, 1.02)	
Unknown	**1.22 (1.02, 1.45)**	0.93 (0.85, 1.01)	**0.89 (0.81, 0.96)**	1.01 (0.92, 1.12)	**1.06 (1.04, 1.07)**	
Insured						0.1064
Private	ref	ref	ref	ref	ref	
Public	1.12 (0.98, 1.28)	0.98 (0.92, 1.04)	**0.93 (0.89, 0.97)**	1.00 (0.96, 1.04)	1.00 (0.97, 1.03)	
Other	0.95 (0.75, 1.20)	1.01 (0.91, 1.12)	0.99 (0.92, 1.06)	0.97 (0.92, 1.04)	1.00 (0.96, 1.04)	

Notes:
PR: prevalence ratio; CI: confidence interval
[a] a p-value for interaction term between wave and characteristic according to a two-tailed chi-square type 3 analysis. Significant values indicate substantial changes in PRs across waves for a specific characteristic. Full list of p-values in Supplementary Table 11.

Supplementary Table 11. Full parameter estimates from a multivariable Poisson regression of spike protein seropositivity in a SARS-CoV-2 serosurveillance system, adults 18-64, New York City (N=24,653).

	PR (95% CI)	P-value for parameter estimate (two-tailed Z test)	P-value for likelihood ratio test (two-tailed chi-square test)
Wave			<0.0001
Wave 1 (through 30 Aug 2020)	ref		
Wave 2 (through 20 June 2021)	3.72 (3.13, 4.43)	<.0001	
Wave 3 (through 31 Oct 2021)	8.09 (6.85, 9.56)	<.0001	
Wave 4 (through 6 March 2022)	9.18 (7.78, 10.82)	<.0001	
Wave 5 (through 18 July 2022)	9.47 (8.05, 11.14)	<.0001	
Age			0.0013
18-44 years	1.26 (1.09, 1.46)	0.0018	
45-64 years	ref	0.0035	
Sex			0.8849
Female	ref		
Male	1.01 (0.86, 1.19)	0.8846	
Race/ethnicity			<0.0001
Asian Pacific Islander	0.67 (0.51, 0.89)	0.0063	
Black	1.27 (0.95, 1.69)	0.1071	
white	ref		
Other	1.55 (1.33, 1.81)	<.0001	
Unknown	1.22 (1.02, 1.45)	0.0278	
Insured			0.1847
Private	ref		
Public	1.12 (0.98, 1.28)	0.1090	
Other	0.95 (0.75, 1.20)	0.6462	
Age*Wave			0.0013
18-44 years*Wave 2	0.76 (0.65, 0.89)	0.0007	

18-44 years*Wave 3	0.76 (0.65, 0.89)	0.0005	
18-44 years*Wave 4	0.78 (0.67, 0.91)	0.0014	
18-44 years*Wave 5	0.81 (0.70, 0.94)	0.0048	
Sex*Wave			0.0035
Male*Wave 2	1.01 (0.85, 1.21)	0.8889	
Male*Wave 3	1.08 (0.91, 1.27)	0.3962	
Male*Wave 4	1.01 (0.86, 1.20)	0.8664	
Male*Wave 5	0.96 (0.82, 1.13)	0.6544	
Race/ethnicity *Wave			<0.0001
Asian Pacific Islander*Wave 2	1.19 (0.88, 1.61)	0.2553	
Asian Pacific Islander*Wave 3	1.59 (1.19, 2.13)	0.0015	
Asian Pacific Islander*Wave 4	1.52 (1.14, 2.03)	0.0043	
Asian Pacific Islander*Wave 5	1.50 (1.13, 2.00)	0.0051	
Black*Wave 2	0.83 (0.61, 1.12)	0.2298	
Black*Wave 3	0.71 (0.53, 0.95)	0.0196	
Black*Wave 4	0.72 (0.54, 0.97)	0.0280	
Black*Wave 5	0.80 (0.60, 1.07)	0.1287	
Other*Wave 2	0.72 (0.61, 0.85)	0.0001	
Other*Wave 3	0.61 (0.52, 0.72)	<.0001	
Other*Wave 4	0.62 (0.53, 0.73)	<.0001	
Other*Wave 5	0.64 (0.55, 0.75)	<.0001	
Unknown*Wave 2	0.76 (0.63, 0.92)	0.0060	
Unknown*Wave 3	0.73 (0.60, 0.88)	0.0014	
Unknown*Wave 4	0.83 (0.68, 1.02)	0.0746	
Unknown*Wave 5	0.87 (0.73, 1.03)	0.1146	
Insured*Wave			0.1064
Public*Wave 2	0.87 (0.75, 1.01)	0.0731	
Public*Wave 3	0.83 (0.72, 0.96)	0.0123	
Public*Wave 4	0.89 (0.78, 1.03)	0.1246	
Public*Wave 5	0.89 (0.78, 1.03)	0.1181	

Other*Wave 2	1.07 (0.83, 1.38)	0.6171	
Other*Wave 3	1.04 (0.82, 1.33)	0.7378	
Other*Wave 4	1.03 (0.81, 1.31)	0.8154	
Other*Wave 5	1.05 (0.83, 1.33)	0.6662	

Supplementary Table 12. Multivariable Poisson regression of spike protein seropositivity among the vaccinated in a SARS-CoV-2 serosurveillance system, New York City (N=8,897).

	Wave 2 (January 11 through 20 June 2021)	Wave 3 (through 31 Oct 2021)	Wave 4 (through 6 March 2022)	Wave 5 (through 18 July 2022)
	PR (95% CI)	PR (95% CI)	PR (95% CI)	PR (95% CI)
Age				
18-44 years	**1.18 (1.11, 1.25)**	**1.13 (1.08, 1.17)**	**1.06 (1.02, 1.11)**	**1.07 (1.03, 1.10)**
45-64 years	1.04 (0.98, 1.10)	1.01 (0.97, 1.06)	1.02 (0.97, 1.06)	**1.04 (1.00, 1.08)**
65+ years	ref	ref	ref	ref
Sex				
Female	ref	ref	ref	ref
Male	1.00 (0.95, 1.05)	**1.04 (1.00, 1.08)**	1.03 (0.99, 1.07)	**0.95 (0.92, 0.98)**
Race/ethnicity				
Asian Pacific Islander	1.03 (0.96, 1.11)	1.03 (0.98, 1.07)	0.97 (0.93, 1.03)	1.00 (0.96, 1.04)
Black	1.06 (0.97, 1.16)	1.00 (0.95, 1.05)	1.00 (0.96, 1.05)	0.98 (0.94, 1.01)
white	ref	ref	ref	ref
Other	1.05 (1.00, 1.11)	1.00 (0.97, 1.04)	1.00 (0.96, 1.03)	1.00 (0.97, 1.03)
Unknown	1.03 (0.95, 1.11)	1.05 (0.99, 1.11)	1.01 (0.90, 1.13)	0.93 (0.82, 1.05)
Insured				
Private	ref	ref	ref	ref
Public	0.97 (0.92, 1.02)	0.98 (0.94, 1.01)	0.98 (0.95, 1.01)	1.01 (0.98, 1.04)
Other	0.98 (0.89, 1.07)	1.02 (0.96, 1.08)	1.01 (0.96, 1.06)	1.01 (0.97, 1.05)

Note: Full list of *p*-values in Supplementary Table 13.

Supplementary Table 13. Full parameter estimates from a multivariable Poisson regression of spike protein seropositivity among the vaccinated in a SARS-CoV-2 serosurveillance system, New York City (N=8,897).

	PR (95% CI)	P-value for parameter estimate (two-tailed Z test)	P-value for likelihood ratio test (two-tailed chi-square test)
Wave			<0.0001
Wave 2 (through 20 June 2021)	ref		
Wave 3 (through 31 Oct 2021)	1.13 (1.05, 1.22)	0.0020	
Wave 4 (through 6 March 2022)	1.22 (1.13, 1.33)	<.0001	
Wave 5 (through 18 July 2022)	1.27 (1.18, 1.36)	<.0001	
Age			<0.0001
18-44 years	1.18 (1.11, 1.25)	<.0001	
45-64 years	1.04 (0.98, 1.10)	0.2146	
65+ years	ref		
Sex			0.9748
Female	ref		
Male	1.00 (0.95, 1.05)	0.9748	
Race/ethnicity			0.4095
Asian Pacific Islander	1.03 (0.96, 1.11)	0.4177	
Black	1.06 (0.97, 1.16)	0.2233	
white	ref		
Other	1.05 (1.00, 1.11)	0.0694	
Unknown	1.03 (0.95, 1.11)	0.5333	
Insured			0.4527
Private	ref		
Public	0.97 (0.92, 1.02)	0.2140	
Other	0.98 (0.89, 1.07)	0.6099	
Age*Wave			0.0008
18-44 years*Wave 3	0.96 (0.89, 1.03)	0.2380	

18-44 years*Wave 4	0.90 (0.84, 0.97)	0.0076	
18-44 years*Wave 5	0.91 (0.84, 0.97)	0.0057	
45-64 years*Wave 3	0.97 (0.90, 1.05)	0.4715	
45-64 years*Wave 4	0.98 (0.91, 1.06)	0.5772	
45-64 years*Wave 5	1.00 (0.93, 1.08)	0.9165	
Sex*Wave			0.0002
Male*Wave 3	1.04 (0.98, 1.10)	0.2244	
Male*Wave 4	1.03 (0.97, 1.10)	0.3314	
Male*Wave 5	0.95 (0.90, 1.01)	0.0780	
Race/ethnicity *Wave			0.5875
Asian Pacific Islander*Wave 3	1.00 (0.92, 1.08)	0.9138	
Asian Pacific Islander*Wave 4	0.95 (0.87, 1.03)	0.2146	
Asian Pacific Islander*Wave 5	0.97 (0.90, 1.05)	0.4712	
Black*Wave 3	0.95 (0.85, 1.05)	0.2968	
Black*Wave 4	0.95 (0.86, 1.05)	0.3010	
Black*Wave 5	0.92 (0.84, 1.02)	0.1113	
Other*Wave 3	0.96 (0.89, 1.02)	0.1749	
Other*Wave 4	0.95 (0.89, 1.01)	0.0998	
Other*Wave 5	0.95 (0.90, 1.01)	0.1200	
Unknown*Wave 3	1.02 (0.92, 1.13)	0.6940	
Unknown*Wave 4	0.98 (0.85, 1.13)	0.8106	
Unknown*Wave 5	0.90 (0.78, 1.05)	0.1738	
Insured*Wave			0.6485
Public*Wave 3	1.01 (0.95, 1.08)	0.7424	
Public*Wave 4	1.01 (0.95, 1.08)	0.6575	
Public*Wave 5	1.04 (0.98, 1.11)	0.1586	
Other*Wave 3	1.04 (0.94, 1.16)	0.4483	
Other*Wave 4	1.03 (0.93, 1.15)	0.5521	
Other*Wave 5	1.03 (0.94, 1.14)	0.5090	

Supplementary Table 14: Point estimates shown within Figure 3. Mean values with bootstrapped confidence intervals are presented for antibody prevalence described in Figure 3A. Geometric mean values and bootstrapped 95% confidence intervals for endpoint titer described in Figure 3B.

Data Range	Measurement	Vaccination status	Mean value	Confidence interval
July 6th - July 20th, 2020 (n=661)				
Antibody prevalence	Nucleoprotein	Vaccinated	N/A	N/A
		Unvaccinated	18.61%	(15.58%, 21.34%)
	Spike	Vaccinated	N/A	N/A
		Unvaccinated	22.39%	(20.04%, 24.93%)
Endpoint titer	Nucleoprotein	Vaccinated	N/A	N/A
		Unvaccinated		(39.25, 47.8)
	Spike	Vaccinated	N/A	N/A
		Unvaccinated		(74.92, 88.19)
February 15th - March 1st, 2021(n=497)				
Antibody prevalence	Nucleoprotein	Vaccinated	15.09%	(5.66%, 26.42%)
		Unvaccinated	23.20%	(19.59%, 27.25%)
	Spike	Vaccinated	51.58%	(42.11%, 62.11%)
		Unvaccinated	30.64%	(27.09%, 33.93%)
Endpoint titer	Nucleoprotein	Vaccinated	36.06	(27.76, 48.71)
		Unvaccinated	47.2	(42.24, 54.23)
	Spike	Vaccinated	383.8	(232.27, 650.13)
		Unvaccinated	95.21	(85.51, 104.71)
June 1st - June 14th, 2021(n=610)				
Antibody prevalence	Nucleoprotein	Vaccinated	56.73%	(51.26%, 62.55%)
		Unvaccinated	58.51%	(53.13%, 63.89%)
	Spike	Vaccinated	86.62%	(83.73%, 89.33%)
		Unvaccinated	63.08%	(59.49%, 66.66%)
Endpoint titer	Nucleoprotein	Vaccinated	66.97	(58.75, 77.32)
		Unvaccinated	81.3	(70.93, 93.41)
	Spike	Vaccinated	1390.59	(1172.2, 1651.96)
		Unvaccinated	292.82	(254.1, 341.19)
August 16th – August 30th, 2021(n=758)				
Antibody prevalence	Nucleoprotein	Vaccinated	54.57%	(50%, 59.38)
		Unvaccinated	53.51%	(47.95%, 58.77%)
	Spike	Vaccinated	86.06%	(83.38%, 88.59%)

		Unvaccinated	62.09%	(57.84%, 65.69%)
Endpoint titer	Nucleoprotein	Vaccinated	72.14	(64.52, 81.21)
		Unvaccinated	67.62	(60.12, 75.86)
	Spike	Vaccinated	1059.25	(922.57, 1221.8)
		Unvaccinated	284.45	(243.78, 331.13)
May 23rd - May 30th, 2022 (n=592)				
Antibody prevalence	Nucleoprotein	Vaccinated	44.82%	(39.90%, 49.75%)
		Unvaccinated	52.69%	(45.70%, 59.69%)
	Spike	Vaccinated	95.81%	(93.60%, 97.54%)
		Unvaccinated	92.47%	(88.71%, 96.24%)
Endpoint titer	Nucleoprotein	Vaccinated	57.41	(50.94, 65.15)
		Unvaccinated	78.49	(64.65, 98.15)
	Spike	Vaccinated	14213.47	(11641.26, 16974.62)
		Unvaccinated	6007.59	(4415.7, 8165.82)
Aug 21st – Oct 2nd, 2023 (n=595)				
Antibody prevalence	Nucleoprotein	Vaccinated	35.12%	(30.51%, 59.49%)
		Unvaccinated	50.97%	(43.69%, 58.25%)
	Spike	Vaccinated	97.97%	(96.45%, 99.23%)
		Unvaccinated	96.11%	(93.68%, 98.54%)
Endpoint titer	Nucleoprotein	Vaccinated	59.37	(52.32, 68.19)
		Unvaccinated	77.7	(65.49, 91.96)
	Spike	Vaccinated	N/A	N/A
		Unvaccinated	N/A	N/A

Supplementary Table 15: Full expanded P-values show within Tigure 3. Comparisons labeled as Chi-squared are shown within Figure 3a while all T tests correspond to Figure 3b. All comparisons are between nucleoprotein and spike values shown within the graph.

Date range	Grouping	Test	P-value
**July 6TH - July 20TH, 2020 (N=661)**			
	Vaccinated	Chi-squared	N/A
		T-test	N/A
	Unvaccinated	Chi-squared	0.0773
		T-test	$1.89x10^{-19}$
**February 15TH - March 1ST, 2021 (N=497)**			
	Vaccinated	Chi-squared	0.0004
		T-test	$1.99x10^{-09}$

	Unvaccinated	Chi-squared	0.0054
		T-test	1.71×10^{-15}
**June 1ST - June 14TH, 2021 (N=610)**			
	Vaccinated	Chi-squared	2.09×10^{-11}
		T-test	1.44×10^{-89}
	Unvaccinated	Chi-squared	0.2680
		T-test	1.67×10^{-25}
**August 16TH – August 30TH, 2021 (N=758)**			
	Vaccinated	Chi-squared	2.63×10^{-25}
		T-test	8.23×10^{-111}
	Unvaccinated	Chi-squared	6.33×10^{-05}
		T-test	4.19×10^{-34}
**May 23RD - May 30TH, 2022 (N=592)**			
	Vaccinated	Chi-squared	2.12×10^{-56}
		T-test	3.07×10^{-246}
	Unvaccinated	Chi-squared	2.17×10^{-17}
		T-test	2.27×10^{-74}
**August 21ST – October 2ND, 2023 (N=595)**			
	Vaccinated	Chi-squared	3.66×10^{-76}
		T-test	4.04×10^{-252}
	Unvaccinated	Chi-squared	9.09×10^{-25}
		T-test	6.19×10^{-101}
