# Supplementary material for: SARS-CoV-2 serosurvey across multiple waves of the COVID-19 pandemic in New York City between 2020–2023
Source: Nat Commun. 2024 Jul 11;15:5847. doi: 10.1038/s41467-024-50052-2 (PMC11239669; doi:10.1038/s41467-024-50052-2)
Supplement: Supplementary file 3 — Reporting Summary [file 41467_2024_50052_MOESM3_ESM.pdf]

nature portfolio

Corresponding author(s): Florian Krammer

Last updated by author(s): Jun 11, 2024

Reporting Summary

Nature Portfolio wishes to improve the reproducibility of the work that we publish. This form provides structure for consistency and transparency in reporting. For further information on Nature Portfolio policies, see our Editorial Policies and the Editorial Policy Checklist.

Statistics

For all statistical analyses, confirm that the following items are present in the figure legend, table legend, main text, or Methods section.

n/a	Confirmed	
☐	☒	The exact sample size (*n*) for each experimental group/condition, given as a discrete number and unit of measurement
☐	☒	A statement on whether measurements were taken from distinct samples or whether the same sample was measured repeatedly
☐	☒	The statistical test(s) used AND whether they are one- or two-sided<br>*Only common tests should be described solely by name; describe more complex techniques in the Methods section.*
☐	☒	A description of all covariates tested
☐	☒	A description of any assumptions or corrections, such as tests of normality and adjustment for multiple comparisons
☐	☒	A full description of the statistical parameters including central tendency (e.g. means) or other basic estimates (e.g. regression coefficient) AND variation (e.g. standard deviation) or associated estimates of uncertainty (e.g. confidence intervals)
☐	☒	For null hypothesis testing, the test statistic (e.g. *F*, *t*, *r*) with confidence intervals, effect sizes, degrees of freedom and *P* value noted<br>*Give P values as exact values whenever suitable.*
☒	☐	For Bayesian analysis, information on the choice of priors and Markov chain Monte Carlo settings
☒	☐	For hierarchical and complex designs, identification of the appropriate level for tests and full reporting of outcomes
☒	☐	Estimates of effect sizes (e.g. Cohen's *d*, Pearson's *r*), indicating how they were calculated

Our web collection on statistics for biologists contains articles on many of the points above.

Software and code

Policy information about availability of computer code

Data collection	Sample metadata, including collection week, demographics, neighborhood tabulation areas, and insurance information were provided as SQL tables by the honest broker and merged into a single datafile using R version 4.2.2 with the packages dplyr (1.1.1), lubridate (1.9.2), odbc (1.3.4), and DBI (1.1.3). Optical densities of dilution series for antibody titer calculations are collected and stored in Excel.
Data analysis	Data were analyzed in SAS version 9.4 (SAS Institute, Cary NC, USA). Visualizations of antibody prevalence and titers were generated using Matplotlib (3.7.2), Seaborn (0.11.2), Pandas (1.5.3), Numpy (1.26.0), and Scipy (1.11.3) packages within python 3.11.5. Geographical charts, and live data explorer were generated using R version 4.3.2, with the packages sf (1.0.14), DT (0.31), zoo (1.8.12), Hmisc (5.1.1), shiny (1.8.0), scico (1.5.0), ggpubr (0.6.0), bsplus (0.1.4), forcats (1.0.0), ggplot2 (3.4.4), ggbreak (0.1.2), patchwork (1.1.3), lubridate (1.9.3), hrbrthemes (0.8.0), data.table (1.14.10), 155 shinyWidgets (0.8.0), RColorBrewer (1.1.3), shinydashboard (0.7.2), and shinycssloaders (1.0.0). Code is available at doi:10.6084/m9.figshare.25962448.

For manuscripts utilizing custom algorithms or software that are central to the research but not yet described in published literature, software must be made available to editors and reviewers. We strongly encourage code deposition in a community repository (e.g. GitHub). See the Nature Portfolio guidelines for submitting code & software for further information.

Data

Policy information about availability of data

All manuscripts must include a data availability statement. This statement should provide the following information, where applicable:

- Accession codes, unique identifiers, or web links for publicly available datasets
- A description of any restrictions on data availability
- For clinical datasets or third party data, please ensure that the statement adheres to our policy

The dataset and code used for regression models is available on figshare at: https://doi.org/10.6084/m9.figshare.24886953.
All data are available under ImmPort accession # SDY2491. Immport is releasing the data on 6/28 27/24 as part of their quarterly data release.

Research involving human participants, their data, or biological material

Policy information about studies with human participants or human data. See also policy information about sex, gender (identity/presentation), and sexual orientation and race, ethnicity and racism.

Reporting on sex and gender	Records about sex were derived from participant self-report.
Reporting on race, ethnicity, or other socially relevant groupings	Records about race were derived from participant self-report. Differences in antibody titers by race are displayed in the paper, and we contextualize these differences in terms of the policies and population experiences during the pandemic.
Population characteristics	Overall, there are 55,092 individuals sampled between February 9, 2020 and July 18, 2022: 21,075 from urgent care group and 34,017 from the routine care group. Of these 882 were children <18, and 54,210 were adults. In the routine care groups, a plurality of individuals were in the 18-44 year age group (16,499, 49%), a majority female (23,155, 69%), a plurality self-identified as White (14,971, 45%), and about half had private insurance (16,386, 49%).
Recruitment	Participants were patients at Mount Sinai.
Ethics oversight	The study protocol HS 20-00308 was reviewed by the Mount Sinai Health System Institutional Review Board, Icahn School of Medicine at Mount Sinai, and it was exempt from human research as defined by regulations of the Department of Health and Human Services (45 CFR 46. 104).

Note that full information on the approval of the study protocol must also be provided in the manuscript.

Field-specific reporting

Please select the one below that is the best fit for your research. If you are not sure, read the appropriate sections before making your selection.

☒ Life sciences ☐ Behavioural & social sciences ☐ Ecological, evolutionary & environmental sciences

For a reference copy of the document with all sections, see nature.com/documents/nr-reporting-summary-flat.pdf

Life sciences study design

All studies must disclose on these points even when the disclosure is negative.

Sample size	This study was started at an early phase of the COVID-19 pandemic. Precision in modeling was not yet available to estimate what the eventual levels of seropositivity would be. We present statistical analyses by epidemiological wave. Each wave had several thousand individuals at minimum (ranging from n=2,849 in wave 4 to 13,562 in wave 2). These are adequate for our statistical design, which is a multivariable analysis with about 4 covariates in an interaction term with wave.
Data exclusions	No particular data exclusions, although we limited most analyses to individuals with a routine care appointment. The final multivariable model included covariates. This is already mentioned in the methods ("Based on a priori considerations, we include age, sex, race/ethnicity, and insurance status in our multivariable models")
Replication	Code for this study is publicly available. Multiple data analysts worked on this project and shared code to mitigate any issues with reproducibility.
Randomization	We did not have any randomization.
Blinding	We did not blind results.

Reporting for specific materials, systems and methods

We require information from authors about some types of materials, experimental systems and methods used in many studies. Here, indicate whether each material, system or method listed is relevant to your study. If you are not sure if a list item applies to your research, read the appropriate section before selecting a response.

Materials & experimental systems

n/a	Involved in the study
☐	☒ Antibodies
☒	☐ Eukaryotic cell lines
☒	☐ Palaeontology and archaeology
☒	☐ Animals and other organisms
☒	☐ Clinical data
☒	☐ Dual use research of concern
☒	☐ Plants

Methods

n/a	Involved in the study
☒	☐ ChIP-seq
☒	☐ Flow cytometry
☒	☐ MRI-based neuroimaging

Antibodies

Antibodies used	IgG (Fab-specific) horseradish peroxidase (HRP) antibody (produced in goat; Sigma-Aldrich, Cat#A0293, RRID: AB_257875). Working dilution: 1:3000.
Validation	All commercial antibodies were validated by their manufacturers and were titrated in the lab to determine optimal concentration for experimentation. MAb concentrations were standardized based on the assay and were used at the starting concentration described in the methods section.

Plants

Seed stocks	Not applicable
Novel plant genotypes	Not applicable
Authentication	Not applicable
